# Evaluating preservation effects on honeybee gut microbiota inocula

**DOI:** 10.1128/spectrum.02754-25

**Published:** 2025-12-03

**Authors:** Kang Wang, Zixuan Xu, Jinmeng Ma, Ming Zheng, Yi Zhang, Zheguang Lin, Ting Ji

**Affiliations:** 1 College of Animal Science and Technology, Yangzhou University 38043, Yangzhou, China; Brigham Young University, Provo, Utah, USA

**Keywords:** honeybees, gut microbiome, intestinal colonization, storage condition, absolute quantification

## Abstract

The honeybee (*Apis mellifera*) gut microbiota serves as a model for host–microbe studies, as germ-free bees enable a relatively sterile system. Fresh gut homogenates are optimal for colonization, but immediate use may be limited by practical considerations, such as pathogen screening and the need to minimize repeated preparation for animal welfare. In this study, we systematically evaluated how short-term preservation conditions influence the colonization efficiency and structural integrity of the honeybee gut microbiota. Gut homogenates were prepared and either used immediately, refrigerated for one day or three days, or frozen for seven days with or without glycerol, before being fed to germ-free bees. Colonization outcomes were assessed by combining absolute quantification of bacterial 16S ribosomal RNA gene copy numbers with diversity indices and community composition analyses. Survival rates of inoculated bees did not differ significantly among treatments, but bees receiving inocula refrigerated for three days showed reduced gut mass, suggesting compromised microbial activity. Absolute quantification revealed that total bacterial loads were broadly maintained, yet taxon-specific responses varied, with *Gilliamella* showing marked sensitivity to freezing. Discrepancies between relative and absolute measurements highlighted the importance of absolute quantification for accurately evaluating colonization. Overall, inocula retained the ability to establish the characteristic core microbiota under all preservation conditions. Notably, refrigeration for one day preserved community features without any detectable differences from freshly prepared inocula, whereas longer refrigeration and freezing introduced measurable perturbations. These results provide methodological guidance for honeybee microbiota transplantation experiments and underscore the necessity of absolute quantification in microbiome research.

**IMPORTANCE:**

The gut microbes of honeybees are essential for their nutrition, immunity, and overall health. To study these microbes, scientists often use germ-free bees colonized with gut communities from donor bees. Fresh preparations are ideal, but repeated preparation is time-consuming, requires pathogen checks, and may involve sacrificing many bees. In this study, we tested practical preservation methods and found that both refrigerated and frozen samples retained the ability to colonize germ-free bees. However, one day of refrigeration preserved microbial abundance and composition most similar to fresh samples, whereas longer refrigeration and freezing introduced detectable changes. These findings provide researchers with clear guidance for preparing standardized microbial inocula while also minimizing repeated dissection of donor bees, thereby improving both experimental reproducibility and animal welfare.

## INTRODUCTION

Honeybees (*Apis mellifera*) are essential pollinators in agricultural and natural ecosystems, providing critical services for crop production and biodiversity maintenance (1). The honeybee gut microbiota has become a focal point of increasing research interest, given its critical roles in nutrition, immunity, pathogen defense, and the regulation of host behavior (2–4). Adult worker bees harbor a relatively simple but highly specialized gut consortium dominated by *Lactobacillus* Firm-5, *Bombilactobacillus* Firm-4, *Bifidobacterium*, *Snodgrassella alvi*, and *Gilliamella* (5, 6). This stable, host-adapted microbiota not only contributes to bee physiology and resilience but also provides a tractable system that has established honeybees as a valuable model for investigating host–microbiota relationships (7, 8).

Experimental studies of honeybee gut microbiota often rely on germ-free bees, which can be colonized with microbial inocula derived from natural colonies (9). This approach provides a controlled framework to test how specific environmental or experimental factors influence colonization success and community dynamics. A practical challenge in such experiments is the preparation and preservation of inocula. While freshly prepared gut homogenates are considered optimal, immediate use is not always feasible, and short-term storage is frequently required. In practice, additional time is often needed to screen inocula for potential pathogens before administration (3), a step that is critical for avoiding confounding effects on experimental outcomes (10). Moreover, the possibility of reusing standardized inocula across replicate experiments can reduce the need for repeated dissection of donor bees, thereby minimizing animal use and aligning with welfare considerations (11).

Previous studies in other host–microbe systems have shown that storage conditions can significantly alter microbial recovery and skew relative abundance estimates (12–15), but systematic assessments in honeybees are lacking. Moreover, most microbiome studies rely on relative abundance data derived from sequencing, which are inherently compositional and may obscure true changes in microbial load (16–19). The development of absolute quantification methods, such as spike-in–based 16S rRNA sequencing, now allows more accurate assessments of microbial colonization and viability across treatments (20–22).

In this study, we investigated how short-term preservation conditions affect the colonization efficiency and structural integrity of honeybee gut microbiota using germ-free bees as a model. Gut homogenates were subjected to freshly prepared, refrigerated (1 day and 3 days), and frozen (7 days with or without glycerol) treatments, and the resulting colonization outcomes were assessed through a combination of absolute quantification, diversity metrics, and community composition analyses. By systematically comparing these conditions, our work provides methodological insights into the optimal handling of microbial inocula for honeybee microbiota research and highlights the importance of absolute quantification in disentangling true biological effects from compositional artifacts.

## MATERIALS AND METHODS

### Gut microbiota preparation

For the preparation of inocula, three colonies were selected as donors. From each colony, three nurse bees with visibly full guts were collected. The guts were dissected under sterile conditions, pooled, and homogenized with a sterile pestle until no visible tissue fragments remained. The homogenate was then resuspended in 800 µL of sterile phosphate-buffered saline (PBS) and further ground against the tube wall to obtain a uniform suspension.

The crude suspension was briefly centrifuged at low speed to remove coarse debris. The supernatant was carefully aspirated and divided into five 80  µL aliquots, each subjected to a distinct preservation condition. One aliquot was mixed with 20 µL PBS and used immediately as the Fresh (Fresh) group. Two aliquots were each mixed with 20 µL PBS and stored at 4°C for 1 day and 3 days, designated as the Refrigerated 1-day (R_1d) and Refrigerated 3-day (R_3d) groups, respectively. The remaining two aliquots were stored at −80°C for 7 days: one mixed with 20 µL PBS (Frozen 7-day, F_7d) and the other mixed with 20 µL glycerol to yield a final concentration of 20% (vol/vol) glycerol (Frozen 7-day + Glycerol, FG_7d). All inocula were prepared and maintained under sterile conditions until use.

### Rearing of germ-free bees

Three healthy colonies of Western honeybees (*Apis mellifera*) were established and maintained in the laboratory apiary of Yangzhou University (Yangzhou, China). Newly emerged worker bees are generally regarded as nearly germ-free, since they have not yet acquired the characteristic gut microbiota; however, they can subsequently become colonized through multiple routes, including contact with nestmates, hive materials, and food sources. In this study, colonization of germ-free bees was established by manually inoculating them with gut microbiota homogenates prepared from natural colonies. To ensure the synchronous emergence of a large cohort of worker bees, the queen was restricted to a single comb frame 18 days prior to the beginning of the experiment, which resulted in intensive oviposition overnight. The frames with brood combs from three independent colonies were subsequently brought to the laboratory and maintained in an incubator under sterile conditions at 35°C and 90% relative humidity until adult emergence. Dark-eyed, late-stage pupae were carefully collected from capped brood frames and individually placed into sterile 24-well tissue culture plates. The plates were maintained under aseptic conditions to allow adult emergence. The next day, newly emerged, active worker bees were randomly selected and transferred into sterile rearing cages, with 20 individuals allocated per cage. All experiments were performed with three biological replicates.

### Microbiota inoculation

The previously prepared gut homogenate was mixed with an equal volume of sterile 50% (wt/v) sucrose solution (1:1) to prepare the inoculum. Germ-free bees were starved for approximately 5 h to enhance uptake and then orally inoculated with 5 µL of the suspension. Following inoculation, bees were supplied with a sterile pollen patty composed of a 1:1 mixture of 50% sucrose solution and gamma-irradiated ground floral pollen.

Both the preparation of germ-free bees and the inoculation procedure were performed repeatedly on days 2, 4, and 8, each time using inocula prepared under the designated storage conditions (Fresh, R_1d, R_3d, F_7d, and FG_7d) to complete the experimental comparisons.

### Experimental maintenance and sampling

Each group was maintained in sterile plastic cages in an artificial climate incubator at 30°C and 60% relative humidity under constant darkness to simulate in-hive conditions. Dead bees were removed daily to reduce the risk of contamination, and survival was recorded for 10 consecutive days. On day 10, all surviving bees were anesthetized with CO₂ and surface-sterilized by immersion in 75% ethanol, followed by three rinses with sterile distilled water. The entire gut of each bee was then carefully dissected under aseptic conditions, weighed individually to determine gut mass, immediately flash-frozen in liquid nitrogen, and stored at −80°C until further analysis.

### Quantification and structural analysis of the gut microbial community

To quantify both the abundance and community structure of gut microbiota in bees inoculated with preserved microbiota, we employed a spike-in–based absolute quantification 16S rRNA sequencing. For each treatment, three biological replicates were analyzed, and each replicate included seven individually processed bees (*n* = 21 bees per treatment in total). Genomic DNA was extracted from gut samples using the FastDNA SPIN Kit (MP Biomedicals, Santa Ana, CA, USA). The quality of genomic DNA was assessed using agarose gel electrophoresis and Qubit fluorometric quantification to verify integrity and measure concentration, respectively. Absolute quantification sequencing was performed using the Accu16S platform (Genesky Biotechnologies Inc., Shanghai, China), which is based on a spike-in internal standard strategy patented under Chinese National Invention Patent No. ZL201910295389.X. Briefly, artificial spike-in molecules were designed to resemble bacterial 16S rRNA genes by retaining conserved regions while randomizing variable regions with approximately 40% GC content, which shared no sequence identity with any nucleotide sequences deposited in public databases. Nine concentration-gradient standards, selected based on preliminary experiments to cover the expected abundance range, were incorporated into each DNA sample prior to amplification to enable absolute quantification. The bacterial 16S rRNA genes and spike-in templates were amplified in parallel using primers 341F and 805R, and PCR products were purified with AMPure XP beads, quantified with Qubit, and pooled in equimolar amounts to construct barcoded libraries following Illumina’s standard protocol. Sequencing was performed on an Illumina NovaSeq 6000 platform, using a paired-end 2 × 250 bp configuration.

Sequence data were processed with the QIIME2 pipeline (23). After trimming primers and adapters with Cutadapt, reads were denoised, filtered for chimeras, and resolved into amplicon sequence variants (ASVs) using DADA2 (24). Taxonomic classification of representative ASVs was performed with a naïve Bayes classifier trained on a honeybee gut reference database, with an 80% confidence threshold. Relative taxon abundances were obtained from the ASV table, and absolute abundances were calculated by scaling relative values according to the total 16S rRNA gene copy numbers estimated from the spike-in controls. Microbial community differences among treatments were analyzed with unweighted/weighted dissimilarity, visualized through non-metric multidimensional scaling (NMDS), and statistically tested using PERMANOVA. In addition, alpha diversity indices (Shannon, Simpson, and Pielou’s evenness) were calculated to compare within-sample diversity across groups.

### Statistical analyses

The Kruskal–Wallis test with Bonferroni correction for multiple comparisons was used to evaluate bacterial load, whole gut mass, alpha diversity indices, and the relative abundances of core microbial taxa among groups. Differences in community composition were assessed using PERMANOVA implemented in the vegan package. Survival data were analyzed using the Kaplan–Meier method, and survival curves were compared with the log-rank test. Statistical significance was defined as *P* < 0.05.

## RESULTS

### Survival and gut mass of inoculated bees

**Fig 1:**
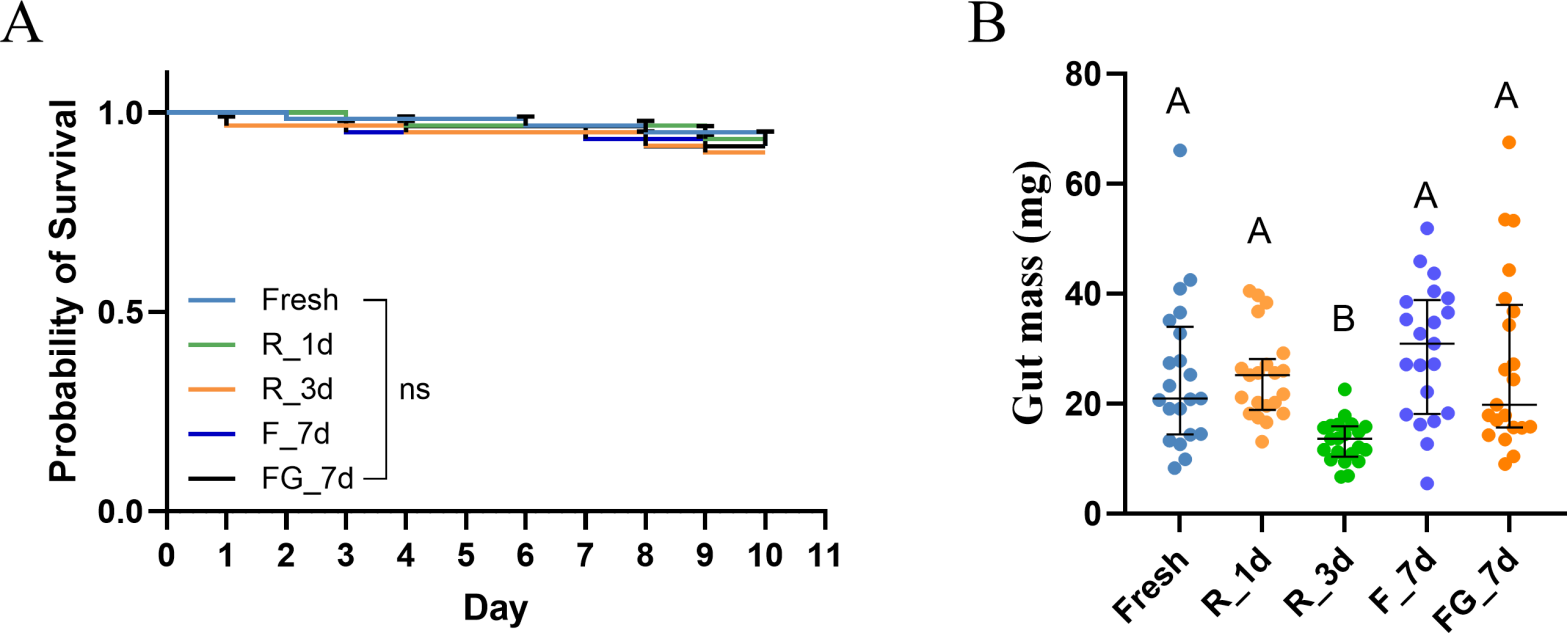
Survival and gut mass of germ-free bees inoculated with microbiota preserved under different conditions. (**A**) Kaplan–Meier survival curves of bees inoculated with freshly prepared gut homogenates (Fresh), inocula refrigerated at 4°C for 1 day (R_1d) or 3 days (R_3d), and inocula frozen at −80°C for 7 days without (F_7d) or with glycerol as cryoprotectant (FG_7d), *n* = 60. No significant differences in survival were detected among groups (log-rank test). (**B**) Gut mass (mg) of bees at day 10 post-inoculation. Bees in the R_3d group showed significantly reduced gut mass compared with all other groups (Kruskal–Wallis test with Bonferroni correction, *P* < 0.01; *n* = 21). Values are presented as mean ± SD. No significant differences were detected among groups (log-rank test; ns, not significant; *P* > 0.05).

The survival of germ-free bees inoculated with gut microbiota preserved under different conditions did not differ significantly among treatments over the 10-day experimental period, as shown by Kaplan–Meier survival curves and log-rank tests (*P* > 0.05, Fig. 1A). Overall, survival rates remained above 80% across all groups.

By contrast, gut mass varied significantly across treatments (Kruskal–Wallis test, *P* < 0.05; Fig. 1B). Post-hoc pairwise comparisons with Bonferroni correction revealed that bees inoculated with microbiota preserved at 4°C for 3 days (R_3d) had a significantly reduced gut mass (13.17 ± 3.81 mg) compared with all other groups (Fresh = 25.29 ± 13.61 mg, R_1d = 25.09 ± 7.95 mg, F_7d = 29.55 ± 12.08 mg, FG_7d = 27.31 ± 16.25 mg; *P* < 0.01), corresponding to roughly 50% of the average gut mass observed in the other groups. No other significant differences were detected among the Fresh, R_1d, F_7d, and FG_7d treatments after correction for multiple comparisons (*P* > 0.05).

### Bacterial loads and community composition

**Fig 2:**
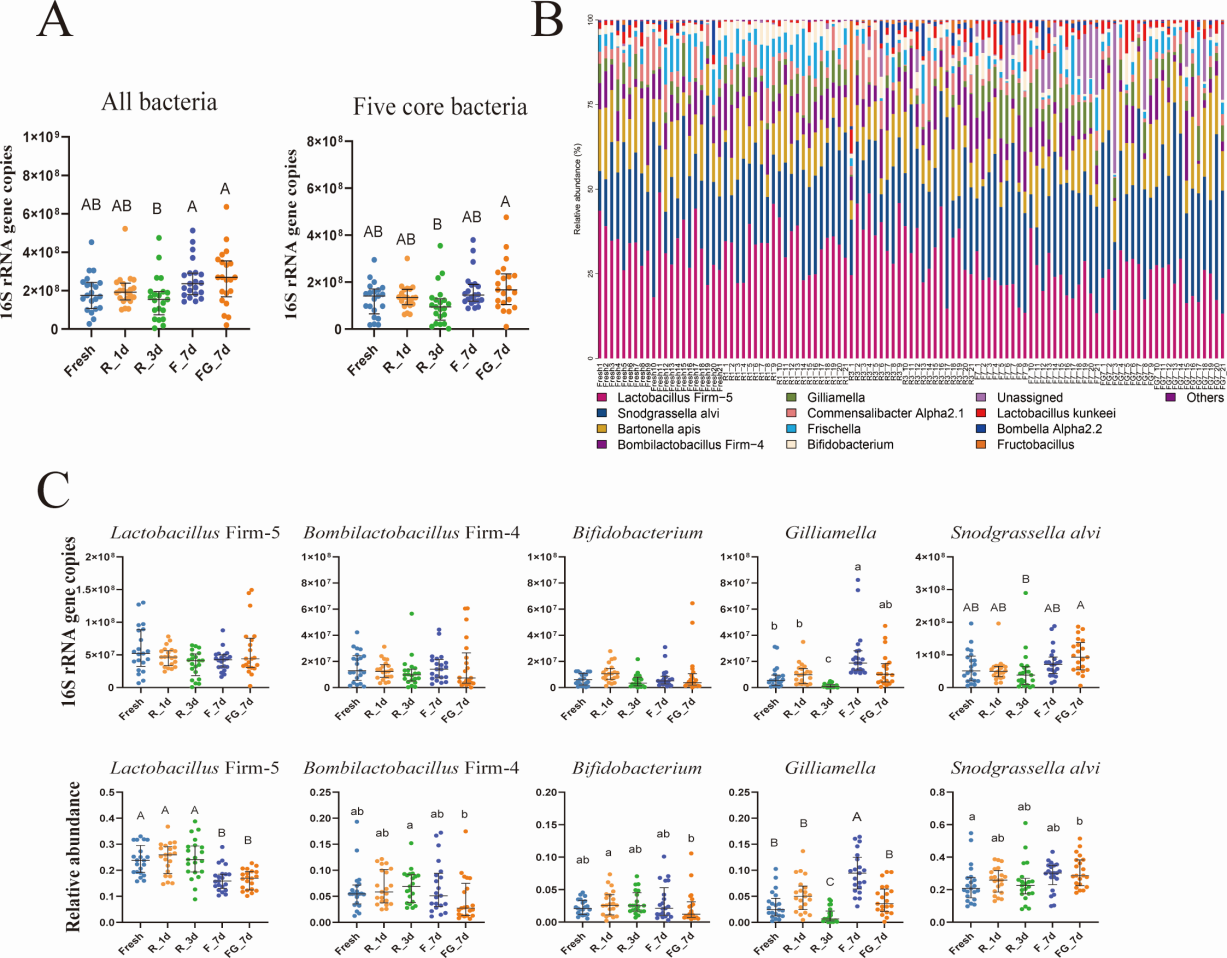
Absolute and relative abundances of core honeybee gut bacterial taxa across preservation treatments. (**A**) Total bacterial load and combined load of the five core taxa based on 16S rRNA gene copy numbers, *n* = 21. (**B**) Gut community composition at the genus level across treatments. (**C**) Absolute abundances (upper panel) and relative abundances (lower panel) of the five core taxa under different preservation conditions. Significant differences relative to Fresh or among treatments were assessed using the Kruskal–Wallis test with Bonferroni correction.

Absolute quantification of gut bacterial loads, based on 16S rRNA gene copies, indicated that both the total bacterial abundance and the combined abundance of the five core gut taxa varied among treatments (Kruskal–Wallis test, *P* < 0.05; Fig. 2A). However, post-hoc pairwise comparisons with Bonferroni correction showed that none of the treatment groups differed significantly from the Fresh control. This result suggests that, although certain differences were observed among preservation methods, overall bacterial loads remained broadly comparable to those of freshly prepared inocula. All previously reported core gut bacteria were detected in every group and collectively accounted for the dominant fraction of the community, although their relative contributions varied among treatments (Fig. 2B). Focusing on comparisons with the Fresh control, absolute 16S rRNA gene copies of *Lactobacillus* Firm-5, *Bombilactobacillus* Firm-4, and *Bifidobacterium* did not differ under any preservation treatment (Kruskal–Wallis test, *P* > 0.05; Fig. 2C, upper panel). Although *Gilliamella* and *Snodgrassella alvi* showed noticeable variation across treatments (Kruskal–Wallis test, *P* < 0.05), post-hoc pairwise comparisons with Bonferroni correction revealed that, relative to the Fresh control, only the F_7d group differed significantly (Kruskal–Wallis test, *P* < 0.05). The other apparent differences were primarily observed among treatment groups themselves, such as between R_3d and FG_7d, rather than in comparison with the Fresh control (Kruskal–Wallis test, *P* > 0.05).

Analysis of relative abundances further highlighted treatment-specific shifts in certain taxa (Fig. 2C, lower panel), with some taxa showing greater variation in relative than in absolute abundance. *Lactobacillus* Firm-5 was significantly reduced in both frozen groups (F_7d and FG_7d) compared with the Fresh control (Kruskal–Wallis test, *P* < 0.05), whereas R_1d and R_3d did not differ from the Fresh group (Kruskal–Wallis test, *P* > 0.05). *Bifidobacterium* was significantly increased in FG_7d compared with the Fresh group (Kruskal–Wallis test, *P* < 0.05), while *Bombilactobacillus* Firm-4 showed no significant difference from the control under any preservation condition (Kruskal–Wallis test, *P* > 0.05). For *Gilliamella*, only the F_7d group exhibited a higher relative abundance than Fresh (Kruskal–Wallis test, *P* < 0.05), whereas R_3d was lower but not significantly different after correction (Kruskal–Wallis test, *P* > 0.05). *Snodgrassella alvi* abundance in FG_7d was slightly elevated (Kruskal–Wallis test, *P* > 0.05), but other treatments were comparable to Fresh group (Kruskal–Wallis test, *P* < 0.05). Notably, absolute quantification confirmed that total bacterial loads remained largely stable across groups, indicating that the shifts observed in relative abundance did not necessarily reflect changes in absolute copy numbers. Interestingly, *Fructobacillus*, a non-core member of the honeybee gut microbiota, exhibited a pronounced increase in absolute abundance in the R_3d group compared with other treatments (Kruskal–Wallis test, *P* < 0.0001; Fig. S1).

### Gut microbiota diversity and community structure

**Fig 3:**
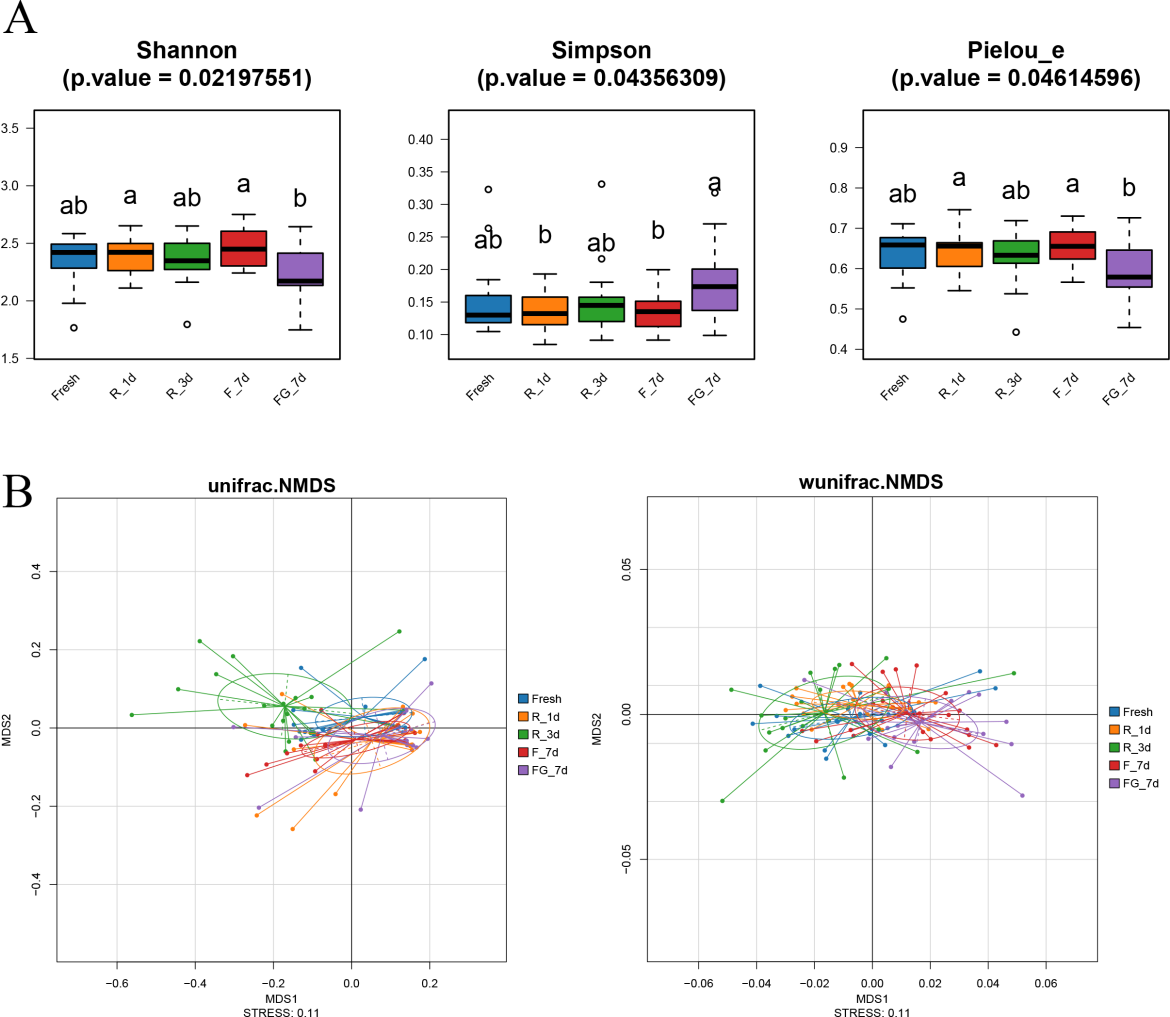
Diversity and structural changes in gut microbiota under different preservation treatments. (**A**) Alpha diversity indices (Shannon, Simpson, and Pielou’s evenness) of microbial communities. Significant differences among treatments were tested by the Kruskal–Wallis test with Bonferroni correction (*P < 0.05). (**B**) Non-metric multidimensional scaling (NMDS) plots based on unweighted/weighted UniFrac dissimilarities, showing partial clustering by treatment. PERMANOVA results confirmed significant effects of preservation on community composition (unweighted UniFrac: *R*² = 0.165, *P* < 0.001; weighted UniFrac: *R*² = 0.246, *P* < 0.001).

To evaluate the effects of microbial preservation on gut microbiota diversity, we analyzed Shannon, Simpson, and Pielou’s evenness indices across groups (Fig. 3A). All three metrics showed significant overall differences among groups (*P*  <  0.05). However, post-hoc comparisons revealed that these differences primarily occurred between preserved groups (e.g., FG_7d vs F_7d or R_1d), rather than between preserved samples and the Fresh group. Notably, the FG_7d group consistently exhibited lower diversity and evenness and higher dominance, suggesting a shift toward a less balanced microbial community structure under the frozen-with-glycerol condition. To further explore whether different preservation conditions altered the overall structure of the gut microbiota, β-diversity was assessed based on both unweighted and weighted UniFrac distances. As shown in the NMDS plots (Fig. 3B), samples from different groups exhibited partially distinct clustering patterns, particularly along the first axis. PERMANOVA analysis confirmed that these differences were statistically significant for both distance metrics (unweighted: *R²*  = 0.165, *P*  <  0.001; weighted: *R²*  = 0.246, *P*  <  0.001). These results indicate that preservation methods significantly impacted the gut microbiota composition, affecting not only the presence or absence of specific taxa but also their relative abundances within the community.

To better understand the group-wise effects, comparisons were evaluated using both presence/absence (unweighted) and abundance-weighted (weighted) beta diversity metrics. Notably, only the R_1d group remained statistically indistinguishable from Fresh in both analyses (Table 1, *P* > 0.05), implying that refrigeration for one day had negligible influence on the gut microbiota. In contrast, significant deviations were detected in R_3d (unweighted) and in both frozen groups (weighted), indicating time- and condition-dependent disruption of microbial community profiles. In summary, all current analytical indicators consistently identified the R_1d group as the only treatment that was not statistically different from the Fresh control.

**TABLE 1:** Pairwise PERMANOVA between fresh and preserved groups

Comparison	Unweighted UniFrac			Weighted UniFrac		
	F.Model	R2	Pr(>F)	F.Model	R2	Pr(>F)
Fresh vs R_1d	1.712144	0.041047	0.1268	2.254764	0.053361	0.0935
Fresh vs R_3d	7.81862	0.163506	0.0002	1.853259	0.04428	0.1384
Fresh vs F_7d	1.111085	0.027026	0.2866	8.748655	0.179465	0.0009
Fresh vs FG_7d	2.001751	0.047659	0.1001	9.792957	0.196674	0.0003

## DISCUSSION

In this study, we systematically evaluated the impact of short-term storage conditions on the colonization efficiency and community integrity of honeybee gut microbiota using germ-free bees as a model. By integrating absolute 16S rRNA quantification with diversity and compositional analyses, we demonstrated that the preservation method influenced both microbial abundance and structure, albeit to varying extents. Among the tested treatments, refrigeration for one day was the only condition that remained statistically indistinguishable from freshly prepared inocula across multiple indicators, whereas prolonged refrigeration and freezing treatments induced detectable perturbations.

At the host level, no differences in survival were detected among groups, suggesting that microbial preservation did not induce strong lethal effects within the ten-day rearing period. However, bees receiving inocula refrigerated for three days exhibited significantly reduced gut mass. Reduced gut size has been linked to incomplete microbial colonization and impaired nutrient assimilation, as gut bacteria contribute to digestion and metabolic provisioning (25–27). In *Drosophila*, colonization by symbiotic bacteria promotes epithelial cell renewal and intestinal homeostasis (28–30). Certain taxa, such as *Acetobacter*, have been shown to stimulate the epidermal growth factor (EGF) pathway, thereby enhancing stem cell proliferation and influencing gut structure (31, 32). In addition, bacterial metabolites, such as acetate, modulate systemic insulin/IGF signaling, linking microbial colonization to host growth, metabolism, and even lifespan regulation (33, 34). In honeybees, studies have shown that gut microbiota colonization increases honeybee gut weight by upregulating developmental genes enriched in signaling pathways such as Wnt and FoxO (35). One possible explanation for the reduced gut mass observed in the R_3d group is the altered microbial composition. Notably, the relative abundance of non-core taxa, such as *Fructobacillus*, increased under this condition. Although typically regarded as fructophilic lactic acid bacteria, *Fructobacillus* has also been detected at high abundance in diseased or stressed colonies, including honeybees suffering from jujube flower disease (36), and is therefore considered a potential indicator of dysbiosis. On the other hand, the reduced gut mass in the R_3d group may also be associated with the decline of the dominant symbiont *Gilliamella*. As a keystone member of the honeybee gut community, *Gilliamella* plays critical roles in carbohydrate breakdown, nutrient assimilation, and the promotion of gut tissue development (37–39). Recent studies reported that *Gilliamella* metabolizes host-derived substrates, such as glucuronate and ascorbate, into glucose, pyruvate, and D-xylulose-5P, thereby promoting lipogenesis. In general, enhanced lipid synthesis is associated with increased body weight, suggesting a potential link between gut symbiont metabolism and host weight gain, although direct measurements of body weight were not included in that study. This is consistent with previous reports showing that an increased abundance of *Gilliamella* in the gut was closely associated with host body weight gain and metabolic disorders under high-fat dietary conditions (40). Thus, a reduction in its abundance may compromise metabolic provisioning and intestinal development, which might underlie the observed decrease in gut mass. Nevertheless, this interpretation remains speculative and should be confirmed by future functional assays directly assessing microbial activity and nutrient assimilation.

We observed instances where trends derived from relative abundance conflicted with those from absolute quantification. This inconsistency arises from the compositional nature of proportional data. Because relative abundances are close to a constant sum, any expansion of stress-tolerant taxa after preservation can inflate their proportions and concomitantly depress the proportions of other taxa even when their absolute 16S copy numbers are unchanged. Absolute, spike-in–anchored estimates therefore provide a more direct measure of colonization success. For example, *Lactobacillus* Firm-5 showed little change in absolute copy number across treatments, yet its relative abundance appeared reduced in frozen groups. This discrepancy reflects not a true decline in Firm-5 but rather the proportional inflation of other taxa, including non-core members and environmentally opportunistic bacteria that expanded under preservation stress. Recent studies have investigated how commonly used preservatives and short-term storage conditions influence microbiome measurements when assessed by both relative and absolute approaches (16, 41). The results showed that preservation methods can differentially affect taxonomic profiles, and that absolute quantification is essential for distinguishing true changes in microbial abundance from compositional artifacts. These insights are consistent with our observation that relative and absolute results did not always align across treatments (22), emphasizing the importance of incorporating absolute abundance data when evaluating the effects of preservation on gut microbiota colonization.

### Conclusion

This study demonstrates that preservation methods exert distinct effects on the colonization and community structure of honeybee gut microbiota. Although all core gut bacterial taxa were successfully detected across preservation conditions, reflecting the retained capacity of inocula to establish the characteristic microbiota, short-term refrigeration for one day preserved microbial abundance and composition most similar to freshly prepared inocula, whereas longer refrigeration and freezing caused measurable shifts at both the host and microbial levels. Moreover, the discrepancy observed between relative and absolute measurements underscores the necessity of incorporating absolute quantification to accurately interpret colonization outcomes. Taken together, these findings provide methodological guidance for honeybee microbiota research, indicating that freshly prepared or briefly refrigerated inocula should be prioritized to ensure reliable and reproducible experimental results.

## Data Availability

Raw sequencing data are publicly available in the Genome Sequence Archive at the China National Genomics Data Center under accession number CRA029396.

## SUPPLEMENTAL MATERIAL

Figure S1 spectrum.02754-25-s0001.docx Absolute abundance of *Fructobacillus*.

ASM does not own the copyrights to Supplemental Material that may be linked to, or accessed through, an article. The authors have granted ASM a non-exclusive, world-wide license to publish the Supplemental Material files. Please contact the corresponding author directly for reuse.
