# Supplementary material for: Evaluating preservation effects on honeybee gut microbiota inocula
Source: Microbiol Spectr. 2025 Dec 3;14(1):e02754-25. doi: 10.1128/spectrum.02754-25 (PMC12772278; doi:10.1128/spectrum.02754-25)
Supplement: Figure S1 — Absolute abundance of Fructobacillus. [file spectrum.02754-25-s0001.docx]

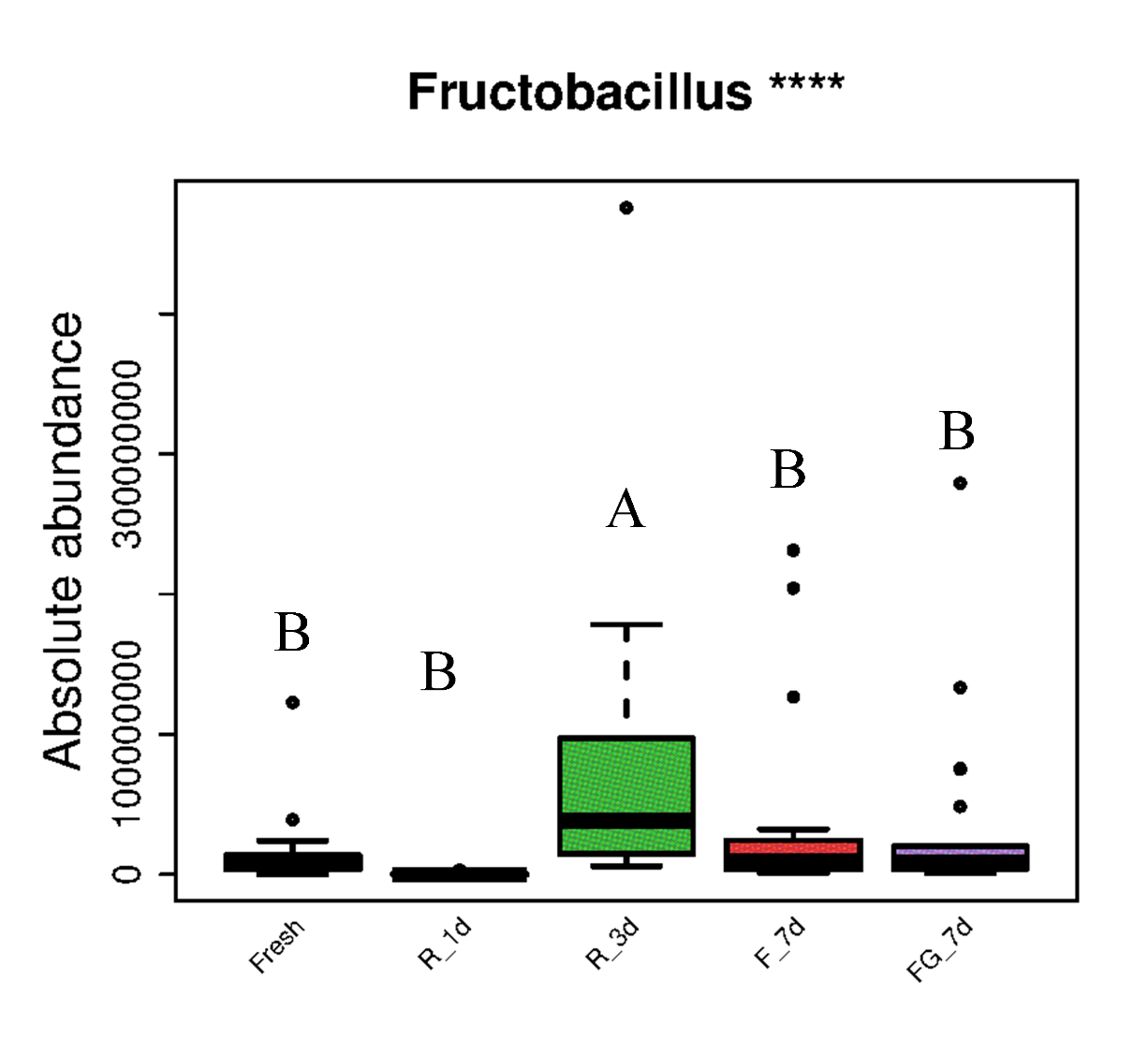


**Supplementary Figure S1**. Absolute abundance of *Fructobacillus* under different preservation treatments. The R_3d group showed a markedly higher abundance (*p* < 0.0001).


